# Above chaos, quest, and restitution: narrative experiences of African immigrant youth’s settlement in Canada

**DOI:** 10.1186/s12889-018-5239-6

**Published:** 2018-03-07

**Authors:** Roberta Lynn Woodgate, David Shiyokha Busolo

**Affiliations:** 1 Rady Faculty of Health Sciences, College of Nursing, University of Manitoba, Winnipeg, MB R3T 2N2 Canada; 2 Faculty of Nursing, University of New Brunswick, Fredericton, NB E3B 5A3 Canada

**Keywords:** Youth, Immigrant, Refugee, Social determinants of health, Settlement, Qualitative research

## Abstract

**Background:**

African Immigrant and refugee youth represent an increasing group of newcomers in Canada. Upon their immigration, youth experience challenges that have the potential to lead to poor health, yet little is known about their settlement journey. Accordingly, this qualitative study examines the settlement journey of African immigrant and refugee youth with a focus on how their experiences were shaped by the social determinants of health.

**Methods:**

We conducted a total of 70 interviews with 52 immigrant and refugee youth (ages 13–29 years) who had arrived in Canada in the preceding six years. Qualitative data was analyzed using Frank’s dialogical narrative analysis approach (Frank AW, Practicing Dialogical Narrative Analysis. In: Varieties of Narrative Analysis, 2016).

**Results:**

Youth experienced different settlement journeys that are described in the three narrative typologies of chaos, quest, and restitution. The chaos narrative of *a long road ahead* revealed the themes of ‘facing challenges’ and ‘still the outsider.’ The quest narrative of *not there yet* was represented by the themes of ‘stepping out of your comfort zone’ and ‘being relentless.’ The theme of ‘supportive environments’ depicted the restitution narrative of *dreams become a reality*. Youth highlighted the impact of social determinants of health in their settlement.

**Conclusion:**

Youth experienced different settlement journeys that were mired in chaos and challenges. However, youth were more likely to experience restitution when they received social support and found a sense of belonging. In future, policies and programs that seek to improve immigrant and refugee youth’s settlement experiences need to be multifaceted, offer more support and promote a sense of belonging.

## Background

Globally, in 2013, roughly 28.2 million international migrants (including refugees) were young people (15–24 years) who moved to countries all around the world including developed ones [1]. In 2011, there were 945,130 young migrants under age 25 living in Canada which represented 9.5% of the population of children and youth in the country [2]. In 2012, youth made about 234,793 (12%) of the immigrants and 23,094 (21%) of the refugees that were admitted to Canada [3]. Of those young migrants who became permanent Canadian residents in 2012, almost half came from the Asia and Pacific Region and 25% came from Africa and the Middle East [2]. In Canada, African immigrant and refugee youth (15–24 years) arrived primarily from Morocco [4].

Youth come to Canada as either convention refugees or immigrants and receive different forms of support. Convention refugees who come to Canada to flee persecution, get support from a sponsor in Canada (privately sponsored refugees), the Canadian government (Government Assisted Refugees) or both [5]. If convention refugees settle in Quebec, they get support from the Quebec government [5]. In contrast, immigrants (Skilled Workers, Provincial Nominees or Canadian Experience Class) come to Canada for non-humanitarian reasons and are generally responsible for their settlement. Usually, Skilled Workers and Provincial Nominees come to Canada based on certain qualities (e.g. work experience) and choose where to settle in the country [6]. Canadian Experience Class immigrants are people who are already residing in Canada as temporary residents but decide to stay in the country permanently [7].

Upon immigration, immigrant and refugee youth arrive with high expectations of finding opportunities to make their lives and that of their families better. Those receiving them in Canada, hope youth will settle, integrate into the society, and have a better future. However, immigrant and refugee youth usually face social determinants of health challenges on arrival and find it difficult to take on their settlement journey [8, 9]. Youth usually experience social exclusion and find themselves vulnerable to mental, emotional, and behavioural challenges [8]. Immigrant and refugee youth are also affected by linguistic challenges [10], socioeconomic difficulties, racism, discrimination, and may find it difficult to access the job market [11, 12]. Such challenges mentioned above can lead to poor health.

While it is evident that immigrant and refugee youth experience challenges in their settlement in Canada, qualitative research examining the settlment journey of African immigrant and refugee youth is in its infancy [13, 14]. For example, Jacquet et al. [13] focused on African immigrant and refugee youth’s intergration in francophone schools, whereas Francis and Yan [14] examined their experiences of accessing services in Vancouver. However, qualitative studies for the most part, have focused on immigrant and refugee youth’s experiences in general [10, 11] with little attention given to African youth. Although the work to date is valuable, more qualitative research is needed that details African immigrant and refugee youth’s experiences of settling in Canada.

Therefore, the aim of this paper is to present research findings that add to our understanding of African immigrant and refugee youth’s settlement experiences in Canada with attention to how their experiences are shaped by social determinants of health. The findings emerged from a qualitative study that examined African immigrant and refugee families’ experiences of coming to and settling in Canada with a focus on their health and social services experiences [15]. Using a narrative analysis approach, the findings presented in this paper specifically addressed the research question: “What was it like to come and settle in Canada?” and focuses on youth participants.

## Methods

### Design

A qualitative research study design using narrative analysis approach was used to examine the settlement journey of African immigrant and refugee youth with a focus on how their experiences were shaped by social determinants of health. The qualitative research study approach allowed youth to present their settlement journey using their own words, stories, and realities and allowed the researchers to situate youth’s narratives within social determinants of health [16].

### Participants

This study took part in the city of Winnipeg, located in the Canadian province of Manitoba. In 2016, Winnipeg had a population of 705,244 people [17]. In 2014, 13,811 permanent residents, 435 Government-Assisted Refugees and 1004 privately sponsored refugees arrived in Winnipeg. About 20% of the immigrants and 57% of the Government Assisted Refugees arrived from Africa and the Middle East while about 92% of privately sponsored refugees came from African countries [18]. Fifty-two immigrant and refugee youth (immigrated in < 6 years) from Africa took part in this qualitative study. Youth were recruited through purposive sampling from social places (e.g. local schools, and churches).

### Data collection

When people tell their stories, they express their reality as they would like to tell it, as well as state what they believe listeners are prepared to hear [19]. Accordingly, youth’s migration and settlement stories were collected by means of unstructured individual interviews. The interviews were carried out from places that were most comfortable to the youth (e.g. at their homes, local libraries, or community centres). Three research assistants trained and supervised by the first author conducted the interviews. Youth were given the option to be interviewed in their language of choice (either in English, Swahili, or French) so that they could express themselves in their most comfortable ways. Trained translators were available to provide translation and back interpretation at the interviews. To facilitate youth telling their stories, we asked the following open-ended question: “Could you please tell me what it was like to settle in Winnipeg?” Use of probes such as “Who has been most helpful to you and your family in settling in Winnipeg?” and “What are some of the challenges you and your family face now?” further contributed to youth telling their stories. The interview questions were developed by the researcher (first author) based on literature and extensive experience of working with youth. Part of the interview questions specifically asked about social determinants of health (e.g. social support and social networks). After every interview, field notes were recorded to provide the context and non-verbal communication of the youth. A total of 70 interviews were conducted with each interview lasting for between 30 and 180 min. While most of the youth took part in one interviews, 18 of them required two interviews to tell their stories.

### Data analysis

Qualitative interviews were digitally recorded and transcribed verbatim. In line with qualitative research, data collection took place concurrently with data analysis. Data from the interviews and associated field notes informed data analysis. Every interview was a distinct story that needed to be examined. Both authors utilized Frank’s dialogical narrative analysis approach to analyze youth’s stories [19]. According to Frank [19], narrative analysis allows the researcher to examine and present what matters to interviewees in ways that those who tell their stories could hear each other and could be heard as a group. The process involved deciding what would make up a story, selecting a story, and examining the story as a whole. We went on to open up the analysis by asking questions about the stories (e.g. how do social determinants of health shape the story). The analysis involved the reading and re-reading of each interview and field notes to provide insight into how youth make meaning of their experiences. Reading multiple times allowed us to sort out the multiple stories embedded in youth’s narratives and decide on what stories to write about in a more practical manner. Systematic reading further involved looking for similarities, differences, and inconsistencies to help draw out the main narratives. We then grouped youth’s stories into three narrative typologies: Chaos, quest, and restitution. To ensure rigour, researchers spent a prolonged period of time engaged with the data and maintained an audit trail [16, 20].

## Results

Our results are presented as follows: a summary of participants’ characteristics followed by youth narratives.

### Participants’ characteristics

Twenty-seven (52%) females and twenty-five (48%) males between ages 13 and 29 years old took part in this study (Table 1). Youth were originally from 18 countries with majority of them immigrating from the Democratic Republic of Congo (DRC) (*n* = 18; 34.6%), Burundi (*n* = 5; 9.6%), Somalia (n = 5; 9.6%), Sudan (*n* = 3; 5.8%), and Kenya (n = 3; 5.8%).

**Table 1:** Demographic profile

Variable		Frequency	Percent
Gender	Female	27	51.9
	Male	25	48.1
Education level	Grade 8 or less	10	19.2
	Some high school	24	46.2
	High school diploma	8	15.4
	College	3	5.8
	Some university	5	9.6
	Bachelor’s degree	1	1.9
	Not in school	1	1.9
Immigration Status (at arrival)	Government assisted refugees	30	57.7
	Permanent residents	15	28.8
	Privately sponsored refugees	2	3.8
	International students	5	9.6
Employment	Yes	18	34.6
	No	34	65.4
Health Rating	Poor	3	5.8
	Fair	3	5.8
	Good	22	42.3
	Very good	8	15.4
	Excellent	16	30.8

### Narratives

The narratives describe the journeys of newcomer youth after migrating to Canada and how their settlement was impacted by social determinants of health. Three narrative typologies related to their journey: (1) chaos; (2) quest; and (3) restitution are presented. Themes for each of the typologies are also presented.

### A long road ahead: The chaos narrative

A long road ahead represents the chaos narrative where youth had multiple problems [19], crystallized by the immigration journey and settlement experiences. One unpleasant situation led to another with life appearing to go nowhere for youth. While efforts by the youth were made to prevent further collapse, their experiences were shaped by social determinants of health that often resulted in inequities in the lives of youth. With no resolution, chaos ensued.

‘A long road ahead’ typically characterizes chaos that occurred primarily during pre-immigration and arrival stages, but tended to decline as youth settle in Canada. A long road ahead was most talked about by 49 (94.2%) youth; 23 (46.9%) females and 26 (53.1%) males. Each youth had multiple chaos narratives. Overall, chaos was influenced by their early life such as homelessness, poverty, and living in refugee camps. Twenty-eight (53.8%) youth experienced war and their stories were similar to those shared by a 20-year old male government assisted refugee who had migrated from Burundi in 2009. He mentioned:

*One day I was coming from school going home and then I saw some soldiers shooting. I saw a big guy with a gun running in my direction, then he stopped and went to hide. The person looked at me, as if he wanted to shoot me but he uttered, “Go, I do not want them to know that I am here.” I was terrified by his look and got so scared when I saw the other guys coming. I did not know where I was. I got traumatized after that. Like I could not talk and then I did not talk to anybody basically, I could not write either.*

Because of such traumas, youth migrated to Canada for safety reasons and “*because it is a better place to find a job, find a better life and great opportunities”* (14-year-old Kenyan male, privately sponsored refugee, 5-years post-migration).

While youth from war-torn countries were relieved to come to Canada, their peers from non-war-torn countries were not as excited. If anything, some were sad to leave their families and loved ones in Africa. Coming to Canada meant breaking from their main sources of social support networks resulting in chaos (Table 2).

**Table 2:** How Social Determinants of Health Affected Immigrant and Refugee Youth Settlement in Canada

Narrative typology	Social determinants of health	How the social determinants of health affected youth settlement
Chaos	Social support	• Post-migration: Limited social support and discrimination especially for youth who immigrated on their own and those with private sponsors. Separations from close families and friends led to chaos.
	Social network	• Post-migration: Limited social networks filled chaos narratives. Youth felt like ‘the outsider,’ discriminated against because they were newcomers, and struggled to fit in established networks.
	Education	• Pre-immigration: Limited opportunities for good education. Interruptions in schooling. • Post-migration: Youth with limited English comprehension faced difficulties of accessing formal education.
	Housing	• Pre-immigration: Experiences of war led to homelessness and living in refugee camps. • Post-migration: Getting decent housing was a challenge. Rent was very expensive for some of the youth.
	Income and its distribution	• Pre-immigration- Poverty. • Post-immigration – Youth struggled with poverty, repayment of immigration fees, and the need to support their families. They found it difficult to make ends meet.
	Employment and working conditions	• Pre-immigration: Unemployment and underemployment. • Post-immigration: Unemployment and underemployment. Youth with limited employment were challenged by limitations in language comprehension, the need to have their academic credentials validated, and questioned whether they were discriminated against because of their religion or ethnicity.
Quest	Social support	• Post-immigration: Youth sought for friendships and mentors to help them find a sense of belonging, overcome discrimination, and settle.
	Social network	• Post-migration: Youth sought for social networks by taking part in sports, community activities, and school clubs.
	Education	• Pre-immigration: Youth and their parents made the decision to migrate to Canada where they could benefit from better educational opportunities. • Post-migration: Youth sought for language instruction, high school and post-secondary education. When they faced challenges in learning, they did not quit but persisted in their quest. Other youth sought newcomer organizations that provide language and educational support to newcomer families.
	Income and its distribution	• Post-migration: Youth with limited family income sought for ways to raise income by working multiple jobs or selling proceeds from their talent work (e.g. artwork).
	Employment and working conditions	• Post-migration: Youth sought for employment opportunities. Some of them sought for multiple jobs to raise enough income.
Restitution	Social support	• Post-immigration: Youth with families in the city and those who made friends with Canadian peers benefitted from their social support. Government Assisted Refugee youth benefitted from orientation and settlement support from immigration counselors.
	Social network	• Post-migration: youth established themselves in social networks in the communities leading to better settlement experiences and feelings of inclusion.
	Education	• Post-migration: Access to good formal education opportunities and making progress towards desired careers.
	Income and its distribution	• Post-migration: Youth appreciated the many opportunities to work and raise enough income to support themselves and their families.
	Employment and working conditions	• Post-migration: Youth had several employment opportunities. Working gave youth a sense of joy, happiness, motivation, and relieved stress.

*Interviewer: What would you say has been like a sad moment for you?*

*Youth: Probably the day I was told that I would be coming to Canada and they told me that only I would be coming, that was a sad moment. I thought everyone like my friends, cousins, dad’s friends, uncles, and my relatives would be coming with me, but it ended up only being me. So yes that was sad.* (13-year-old Chad male, permanent resident, 5-years post-migration).

In as much as migrating to Canada allowed youth to physically move away from places where they faced challenges and danger, they continued to experience dark moments of past struggles in the midst of new ones. Youth from predominantly war-torn countries found it challenging to let go of those past experiences. Their mental health had been affected by early life experiences of trauma and had a ‘long road ahead’ to better health.

*I still have like a lot of things going on in my mind. When I came here, my mind was not really there, like a hundred percent. I had bad memories that I had to try to erase, refresh my mind, and have a brand new start. Sometimes when I have like my dark moments, I have flashbacks that make me go through my past in Africa. I see all the pain and sometimes when I think about that, tears come out of my eyes because it is really painful.* (19-year-old Kenyan male, government assisted refugee, 6-years post-migration).

While youth talked about mental health challenges, only two (3.8%) of them talked about seeking, accessing or receiving mental health care in the city. Youth gave reasons that included lack of awareness and distrust in mental health care approaches in addressing mental health care needs.

Post-migration roadblocks related to the social determinants of health that further compounded youth’s settlement experiences included inadequate educational opportunities, ongoing struggles with building relationships, discrimination, unemployment and underemployment, and unmet socioeconomic needs in general (Table 2). The chaos narratives were further represented by the themes of facing challenges and still the outsider.

#### Facing challenges

Youth arrived in Canada with great expectations and were hopeful that their lives would be successful. Thirty-six (69.2%) youth; 23 males and 13 females expected Canada to be like “paradise” and to be a place where they would go to school, find work, and become “rich” in a short period of time. However, youth’s expectations were different from what they experienced when they arrived. Upon their arrival, youth struggled to settle and their lives were getting out of control.

Youth were challenged by weather conditions, socioeconomic problems, overwhelming responsibilities, and communication difficulties (Table 2) that made their lives difficult. All youth experienced winter for the first time and because of the cold weather, they found it difficult to go to school or work, as well as to take part in health promotion activities.

*The weather is the worst nightmare that an immigrant coming from a tropical country can experience during their stay in Canada. I had to go to school, look for a job in the cold winter, oh sometimes I was like “God if you want to take me, take me now.”* (20-year old Burundi male, government assisted refugee, 5-years post-migration).

*It is a lot harder to be physically active in Canada because you cannot exercise in the wintertime; you barely want to be outside, like you only want to be out in the summertime.* (18-year-old Sudanese female, permanent resident, 6-years post-migration).

Socioeconomic problems created challenges especially for youth who came to Canada by themselves (Table 2). Fourteen (26.9%) youth talked about struggles with finding suitable employment, discrimination, and experiencing financial needs. In their narrations, youth deliberated difficulties of fulfilling employer expectations (e.g. dress code) and feeling discriminated against because of their gender and/or religion which limited their job opportunities.

*Some challenges that we cannot control are like people that do not hire girls who have a hijab. I understand those companies have a certain uniform but I cannot wear it because of my religion. So I cannot work there obviously. However, I can work as a cleaner, in a kitchen, or in a restaurant but employers are scared because if someone puts on a hijab, they think that the hijab will catch fire.* (19-year-old Somali female, permanent resident, 2-years post-migration).

Other youth who migrated with their parents, talked about their family members finding it difficult to secure employment which made it difficult to meet their basic needs. Some of the youth’s parents relied on social assistance from the government, but the monies were not enough. As a result, youth took up jobs to support their families. Youth talked about feeling overwhelmed by working to support their families as a source of chaos narratives (Table 2). Among the young people who came to Canada with their families, four (7.7%) of them sought employment to support their families. Initially, youth felt that supporting their families was a noble task. However, as they continued to work, they quickly learned that it was difficult to work while going to school. Finding a balance was challenging and their health was affected. Youth experienced fatigue, lack of sleep, and stress.

*I think the responsibilities affected my health because I worked seven days all week then when I went to school I was tired. Sometimes I feel depressed when I go there, that is the problem. I do not sleep well because after work I go to study. I do need to be studying but then I do not get good sleep. When I go to school sometimes I feel depressed.* (19-year-old Ethiopia male, privately sponsored immigrant, 2-years post-migration).

Settling in Canada became more challenging because of communication difficulties and youth felt like their lives were trapped. Fourteen (26.9%) youth struggled with language problems and found it difficult to address social determinants of health (Table 2). The youth found learning at school, access to health care, and making social connections challenging.

*Well at the beginning of the year I could not understand the language. Whenever I went to the gym, the teachers kept telling me to do exercises but I did not know what to do. So I sat still. The teachers would come up to me and tell me things to do, but I kept asking other students what he was telling me*. (15-year-old Somali Male, government assisted refugee, 4 years post-migration).

#### Still the outsider

Chaos narratives were evident when youth talked about feeling like an outsider or a stranger. Being an outsider meant that youth felt different from other people and the communities where they lived and as such, made it harder to achieve employment, housing, education, and social inclusion (Table 2). Upon coming to Canada, ten (19.2%) youth found themselves on their own, without settlement workers or sponsors to orient them to the city. The youth felt unwelcomed and did not have a sense of belonging.

Youth experienced social exclusion and felt that perhaps being treated as the outsider was part of the culture in Canada where people do not usually approach newcomers.

*In Canada, we are less welcoming which is unlike in Africa. When you are new in a classroom, teachers may not even introduce you to everybody in the class, and sometimes you can spend a whole day in the classroom without anybody coming to say “hi” to you*. (13-year-old Cameroon female, family sponsored immigrant, 2-years post-migration).

Because there was no one to connect with socially, youth sometimes followed or interacted with people who did not always have good intentions. Youth assumed they could rely on everyone they interacted with. However, sometimes youth were misled and found themselves taking part in behavior (e.g. smoking or drinking alcohol) that could result in poor health. Also, sometimes youth got themselves into problems because of not knowing Canadian practices, laws, and regulations. In essence, being the unaware outsider led youth into more chaos.

*The problem is when you are new in Canada, there are people doing the wrong things, but sometimes we think they are doing the right thing because they live here. We assume they know everything going on here. When they do wrong things, we think that is the right thing. When we do the wrong things, we get into trouble.* (19-year-old Ethiopia male, privately sponsored immigrant, 2-years post-migration).

While most youth in the study felt isolated and received limited to no help in their settlement, they felt that Government Assisted youth had better experiences. Youth in the study believed Government Assisted Refugees were welcomed and received help in their settlement. While being helped to settle was possible, Government Assisted Refugee youth felt the reception was inadequate. By the end of the six-month orientation period, some of them continued to desire more information.

*Like at anonymous newcomer centre, there were some people who helped and I appreciate that help but they were not the most helpful. They only showed us the bank and gave us ideas, and that is what they were paid for. No one could take up your hand and take you places that you do not want to go, or you do not want to be in* (19-year-old Somali Female, privately sponsored immigrant, 2-years post-migration).

### Not there yet: The quest narrative

Quest refers to an extensive search for something that is hard to find or an effort to accomplish something demanding [19]. In our study, 23 (44.2%) youth searched for ways to settle and at the same time address the social determinants of health needed to achieve good health (Table 2). Quest narratives labelled as ‘Not there yet’ represent the time and struggles towards achieving youth’s dreams.

Youth shared stories of their aspirations for higher education and their desires to pursue professional degrees. Some disclosed their desire to become doctors with the aim of going back to African countries to treat the sick. However, in their quest for a medical degree, youth became aware of the time and struggles it would take to complete medical degrees.

*I want to be a doctor because I want to save peoples’ lives, to help them become healthy. However, most of the people that I meet in the medical field tell me that to become a doctor or to be successful in medical studies I have to be good in mathematics. Since then I decided to learn mathematics and I have been trying hard to become a good mathematician.* (13-year-old Cameroon female, privately sponsored immigrant, 2-years post-migration).

Achieving their goals was difficult because youth grew up in countries where opportunities for training and mentorship were limited. Given that youth were pursuing similar goals as their peers in Canada, they felt disadvantaged because social determinants of health (e.g. early life experiences and social support networks) did not favor them. For instance, one of the youth narrated how he would play soccer with non-immigrant peers but was less likely to progress into professional teams. The immigrant youth did not have social support networks to connect him to better teams the way his non-immigrant peers did. His desire to become a professional athlete was difficult to achieve. In fact, he believed it was possible to end up “*staying in the same team for a couple of years till he gives up on soccer.”* (20-year old Burundi male, government assisted refugee, 5-years post-migration).

The quest narratives were further represented by the themes of stepping out of your comfort zones and being relentless.

#### Stepping out of your comfort zone

Quest involved stepping out of one’s comfort zone to pursue one’s desires. Youth talked about being discontent with their situation and seeking ways to address social determinants of health needs. Seven (13.5%) youth gave examples of ‘stepping out of your comfort zone’ that were similar to those shared by the following youth.

According to one of the government assisted refugee male youth, he encountered challenges upon coming to Canada but took unexpected actions to find solutions. The youth found it difficult to locate a place to showcase and sell his artwork. Therefore, he took an unexpected step of finding other immigrant youth with similar struggles and all searched for a place in the city where they were able to showcase and sell their work. Another challenge that the youth encountered was that his mother found it difficult to support them because of unemployment, poor health, discrimination, and language difficulties. The 20-year old youth from Burundi, stepped up and worked multiple jobs to support the family. Instead of sitting back and waiting for his mother to find a way of supporting them he became the breadwinner by stepping out of his comfort zone.

Another 26-year-old male government assisted refugee youth who immigrated from Sudan in 2006 talked about experiencing multiple demands and stepping out of his comfort zone to find solutions. The participant arrived in another province in Canada with his cousin with the hope of living together and supporting each other. However, the two often found themselves in conflict and could not stay together. The youth moved to Manitoba by driving about 3000 km to join his married sister but that did not help. The youth found himself isolated because the sister and her family planned life events, accessed health, and social services without involving him. At the time of the interview, the youth had difficulties accessing services and programs such as health care and gym facilities. Faced with these difficulties, he made the bold decision to live on his own and moved out of the comforts of his sister’s home. He started volunteering at a local college where he could access gym facilities for free, make friends, and benefitted from career mentorship.

Beyond sharing experiences of ‘stepping out of your comfort zones,’ youth went on to suggest that their peers take similar proactive steps. Youth stressed the need for peers to look beyond being content with their status and challenge themselves. Youth believed they had great potential to meet their social determinants of health needs (e.g. employment). Youth suggested that other youth seek better employment opportunities. For example, one 25-year-old female youth who arrived from Nigeria talked about youth taking up homecare positions which could lead them to better paying jobs. She challenged youth to seek appropriate employment even if it is more demanding. She believed that making an effort was more important than sticking to “easy” jobs. She stated:

*I tell them, “Go out there even if the jobs are challenging. Even if you get fired in the first month of working, you should come back and say, ‘At least I tried.’ You need challenges, you need to step out, wake up in the morning thinking that ‘I am not just doing Homecare which everyone is doing.’ Homecare is so easy because all that one does is to take care of people in their homes. You can do more****.”*** (25-year old Nigerian Female, economic class immigrant, 5-years post-migration).

#### Being relentless

To succeed, the youth had to be relentless. In our interviews, seven (13.5%) youth shared experiences of facing difficult settling experiences but were persistent in pursuing their goals. One of the youth shared his experiences of determination in pursuing university education. The youth was seeking undergraduate education from an English-speaking university but encountered hurdles. He was asked to take advanced English courses but on several occasions, he was denied entry into the classes. He did not give up; he persisted in seeking ways to secure an admission. The 20-year-old youth took a lower level English course then enrolled in a more advanced course at a later stage. On several occasions, he failed at the advanced course but kept on working hard until he passed.

*I asked for the English literature class but I was told I could not take it. I asked them again to let me join a regular English class, but they said, “Well based on what you did last semester we do not think that we can put you in a regular English class.” I wanted to go to the class that’s when they let me. That class helped me. Because I passed that class, I was able to continue to the university.* (20-year old Burundi male, government assisted refugee, 4-years post-migration).

Youth with limited English on their part sought for multiple ways to achieve their dreams. The youth resorted to watching television programs and movies and speaking with people who were more proficient in English. The youth were relentless in their quest to learn English.

*I work so hard as I told you. I took the English course and as a result, I can speak fluently with anyone. I was a new person, so I had to learn, I did not have a choice. I took English two years ago. However, taking the course was not enough, so I watched movies, TV and spoke with other students on campus. That helped me so much because I was always around people speaking English.* (26-year-old Moroccan female, economic class immigrant, 5-years post-migration).

Working hard and being relentless appeared to go beyond youth’s personal goals and to involve other family members. For instance, to address socioeconomic needs, youth sought employment, and then helped their siblings and parents find work. In their narrations, they used words like “I have to,” “do what she can do” and “not giving up” to emphasize their resolve. When youth’s parents noticed their children working hard, they were inspired.

*We are working hard. We tell ourselves; “I have to get a job and get my young brother a job as well.” You know, she (mother) sees that we are trying to do something, then she goes on to do what she can do and I am really proud of her for not giving up in spite of her age. She’s planning to do certain things in life and I am encouraging her.* (24-year-old Congolese male, government assisted refugee, 5-years post-migration).

In recounting quest narratives, youth often mentioned clear lessons from their immigration and settling experiences. Most important, youth learned that settling takes time and perhaps because of discrimination, they needed to outclass their peers. Therefore in as much as immigrant and refugee youth may be relentless, they found they needed to be patient in their quest and to work hard.

*I had to learn that everything takes time. It takes a lot of time and you have to learn about the system. You have to study and challenge the people here to prove that you are better to get what you want. Finally, you have to learn new things every minute. (29-year-old Ghanaian Male, privately sponsored immigrant, 4-years post-migration).*

While youth were relentless and found it necessary to work hard, they were cognizant that not every newcomer youth could face the challenges and persist at finding a solution. Youth had been shaped by early life experiences and relied on self motivation and resilience to pursue their goals.

*It depends so much on one’s personality because when I’m going through challenges I know that it’s probably a good thing. When I have a smooth lifestyle, I feel something’s not right so I try to motivate myself that way and I keep going.* (29-year-old Ghanaian male, privately sponsored immigrant, 4-years post-migration).

### Dreams become a reality: The restitution narrative

‘Dreams become a reality’ represents the restitution narrative. The restitution narrative plot depicts the immigration journey where someone arrives in Canada, acculturates to the lifestyle, adapts, and is settled. Youth were experiencing less stress as their goals were being achieved.

*We are not worried about anything since we got everything that we wanted. We are working, going to school, and graduating. All my brothers have graduated and I am almost done with high school. I can say everything that we planned is kind of going the way that we wanted.* (19-year-old Congolese Female, government assisted refugee, 5-years post-migration).

Youth were thankful for their achievements. Beyond the achievements, they had future goals and were hopeful that such goals could be achieved as well.

*I am happy with my life where I am, I am thankful for what I have. I still believe there’s a lot to do, a lot of people that need help. I am willing to sacrifice and do as much as I can (chuckle). I wanted to do human rights, because I’m thinking of going back in Africa in different camps to help a lot of refugees for the rest of my life.* (13-year-old Chad male, economic class immigrant, 5-years post-migration).

These narratives primarily occurred when youth had lived in Canada for an average of 3.7 years. Among the three narrative typologies, restitution narratives were the least talked about. Twenty-nine (55.8%) youth; 14 (48.3%) females and 15 (51.7%) males shared restitution narratives. The restitution narratives were further represented by the theme of supportive environments.

#### Supportive environments

‘Supportive environments’ refers to a theme where youth talked about their plans and expectations being fulfilled because of receiving support. Youth shared restitution narratives where their stories focused on how they felt welcomed and had a sense of belonging. Youth found ethno-cultural communities that were friendly and willing to support them in their settlement. As a result, youth were no longer experiencing the need to address some of the social determinants of health needs (e.g. social exclusion).

*Coming from Kamloops (city in the Canadian province of British Columbia) was a huge step. I saw so many Africans because in Kamloops you could count us. Like there were about five of us. There were so many African people here (Winnipeg, Manitoba) and they were very accommodating. Like they were very friendly, wanting to help and I was like “wow, I think I like this place.” The apartments were cheap; I was like “whoa.”* (25-year-old Nigerian Female, economic class immigrant, 5-years post-migration).

The narratives went on to highlight how youth formed special bonds and were relating well with their friends. Instead of taking part in risk behavior, youth were taking part in health promotion activities with their peers. Instead of being stressed, youth were having “fun.” Ten (19.2%) youth shared restitution narratives that highlighted their connections with peers, having many friends, and taking part in leisure activities (Table 2). In essence, youth were no longer feeling discriminated against.

*It is fun; I have a lot of good friends. I go to their house, they come to my house, and we go watch movies and stuff like that. When we all don’t have homework we go somewhere to skate, play soccer, basketball and stuff like that or watch games, so it is fun.* (13-year-old Chad male, Economic class immigrant, 5-years post-migration).

To other youth, ‘supportive environments’ was more about their access to services (e.g. after school programs) where they could get assistance. There were social, health, and educational programs in the city which youth could access. The availability of health, social, and educational support programs was unlike the situation in their countries of origin. One of the young people who accesses one of the programs talked about getting support at the educational programs. He reflected on how getting such form of support was not possible in Sudan.

*Everything is good here; like if you want to study its good they help you, if you go to [anonymous] they help you with homework whereas in Sudan there’s nothing like that. Nobody is going to help you.* (13-year-old Sudanese male, government assisted refugee, 4-years post-migration).

Accordingly youth made recommendations for parents to take their children to such educational programs where “they *could end up learning something and make friends”* (19-year old Congolese female, Government assisted refugee, 5-years post-migration).

Furthermore, youth talked about their mentors, supportive parents, and personal networks as important players in their restitution narratives (Table 2). The important players provided youth with orientation to the city, provided advice on careers, academic work, ways of overcoming discrimination, and making life choices. Youth could approach them with questions and expect to receive useful advice, motivation and guidance on how to settle. While the people provided support, they also nurtured youth in ways that they could become independent in future.

*When you have connections, you have this person who is like saying, “You can do this, come over here, I know these people who are going to help you.” I have a mentor who’s helping me with my art. So he tells me where to go and how to speak to certain people. He’s showing you how it works, so you can basically figure it out on your own*. (20-year old Burundi male, government assisted refugee, 5-years post-migration).

## Discussion

Our study examined the journey of African immigrant and refugee youth in their settlement in Canada and how their experiences were shaped by social determinants of health (Table 2). We utilized individual interviews which gave youth the opportunity to construct and share stories about their migration and settlement in meaningful ways. Youth’s stories revealed that migration journeys are not linear, each individual story is made up of multiple narratives. Although we present our findings in three different narrative typologies: chaos, quest, and restitution, youth in our study experienced multiple narratives. The stories highlight the need for policies and programs targeting immigrant and refugee social determinants of health challenges to be multifaceted. We present the recommendations on policies and programs in subsequent paragraphs.

Chaos narratives highlight the pre- and post-immigration challenges that were an impediment to youth’s settlement. These challenges were similar to the preflight, flight, and resettlement challenges shared by other newcomer youth [21, 22]. In spite of this awareness, our study reveals that youth continue to experience challenges long after their immigration yet they receive limited support. Similarly, Francis and Yan [14] reported that gaps in newcomer youth services in Vancouver result in unmet settlement needs. Areas that youth could benefit from are better access to health, education services, and financial support systems (e.g. scholarships and loans) that could augment their socioeconomic needs [23]. Also needed, are protective policies that focus on promoting inclusivity, integration, equality, and access to material resources at provincial and national levels [24, 25].

Immigrant and refugee youth shared stories of mental health challenges yet only two (3.8%) of them talked about accessing mental health care services. Youth from predominantly war-torn countries talked about post-traumatic stress experiences that could be detrimental to their settlement. Youth from countries experiencing conflicts are often vulnerable to mental, emotional, and behavioral needs [8, 26], while youth that witness or experience racism and discrimination in Canada may be vulnerable to mental health problems [21]. While mental health needs were talked about, perhaps youth did not consider themselves to have mental illnesses, or were reluctant to seek care [27]. Youth may not have been offered mental health care because immigration processes usually screen out entry for people with mental illnesses. In future, routine mental health services could be offered to immigrant and refugee youth upon their immigration. Regular and yearly medical examinations should include mental health assessments.

On the theme of ‘still the outsider,’ youth talked about being on their own and feeling like strangers. Similarly, war affected refugee youth who took part in a study by Mbabaali [28] expressed desires to feel loved, accepted, and included as members of their new societies. A lack of a sense of belonging, discrimination, or racism can result in loneliness, low self-esteem, and depression [29–31]. On the contrary, developing connections with one’s communities, families, and culture offer opportunities for better health, and settlement [32]. Youth may find a better sense of belonging if they are allowed to speak about their identity, share life stories, and establish peer relationships with youth in Canada [29]. Immigrant and refugee youth may also find a sense of belonging if they access immigration transition centers and receive training from service providers that are culturally competent. On the other hand, service providers may promote a better sense of belonging by developing and implementing policies that promote hiring of staff from the same ethno-cultural backgrounds as immigrant and refugee youth, providing after school programs for the youth, and by purposefully connecting youth with established immigrant communities where information on settlement and support can be shared [23, 33].

On quest narratives, 23 (44.2%) youth shared stories of struggles and taking time to realize their dreams, stepping out of your comfort zone, and being relentless in their aspirations. Similarly, newcomer refugee youth in Toronto had strong aspirations for advanced education in Canada but faced challenging barriers [22]. The youth in Toronto encountered financial, linguistic, and systemic barriers, as well as discrimination. However, they were able to overcome the challenges by getting help from friends, newcomer support organizations, and questioning authority whenever unfavorable decisions were made. In our study, youth with limited forms of support were more likely to share chaos narratives compared with youth with some form of support. Better support in the form of providing orientation, mentorship in trusting, respectful, and inclusive ways could help youth in the pursuit of their dreams [34]. The support could be provided by their teachers, immigrant support workers, and community leaders. Additionally, policies that dispel discrimination need to be developed. Effective policies could focus on creating awareness about discrimination and creating opportunities for immigrant and refugee youth that positively advance their social determinants (e.g. language acquisition or eliminating poverty).

Restitution narratives focused on youth’s stories about realizing their dreams and benefitting from supportive environments. Unlike refugee youth in Toronto who supported their parents to ensure family well-being [35], 29 (55.8%) youth in our study received orientation, mentorship, career, and educational advice from others (e.g. parents, friends, community members). In the wake of increasing immigrant and refugee populations in Canada and other developed countries [1], there is need for better awareness and access to youth’s supportive environments. The findings in our study demonstrate that access to supportive environments can allow youth to realize their dreams and lessen the burden created by social determinants of health needs (e.g education). However, studies from the United States reveal that although supportive environments could result in addressing social determinants of health (e.g. education) and lead to better health, these effects are more likely for Caucasians than for African/black populations [36–38]. Therefore, Assari [39] recommends developing policies that promote income distribution, eliminate discrimination, and barriers to resources. Although supportive environments were talked about in the restitution narratives, youth’s stories were not devoid of other struggles and the need to address other determinants of health. Therefore immigrant and refugee youth need greater awareness and access to supportive mentorship programs that could lead to better settlement and health.

While the findings of our study are useful, the findings need to be considered in light of the following strengths and limitations. We involved a large group of youth which is the key strength of our study. There were 52 youth from 17 African countries whose stories were similar on the narrative typologies. With regards to study limitations, our study evoked memories and narrations of traumatic, emotional, and difficult life experiences that could be painful to remember. We provided youth with resources on places where they could seek counseling support if needed. Another limitation is that we utilized a cross-sectional data collection approach where participants’ narratives were collected at one point in time. Therefore youth’s experiences that might have changed over time were missed. Youth were provided with the opportunity to be interviewed in either English, French, or Kiswahili. By interviewing youth that could communicate in either of these languages, views from youth who preferred to be interviewed in other languages (e.g. Amharic) might have been missed. In future, research that takes into account our limitations could extend our understanding of youth’s settlement experiences.

## Conclusion

Every year, African immigrant and refugee youth make their way to Canada with great expectations of settling down and pursuing their dreams. However, these youth face challenges based on social determinants of health that include limited social support, social networks, access to formal education, access to housing, poverty, discrimination, and unemployment and underemployment. Despite the challenges, youth often take individual steps to address the difficulties. From the youth’s narratives, the challenges are better addressed leading to favorable settlement experiences when others (e.g. parents and mentors) offer support. Importantly, youth make recommendations for policy makers, service providers, newly arrived youth, and community members that could improve their settlement. Youth could benefit from better access to health, educational services, protective policies, and better social support to help them achieve better settlement.
